# Quantitative Pore Characterization of Polyurethane Foam with Cost-Effective Imaging Tools and Image Analysis: A Proof-Of-Principle Study

**DOI:** 10.3390/polym11111879

**Published:** 2019-11-14

**Authors:** Shemmira Yunus, Baah Sefa-Ntiri, Benjamin Anderson, Francis Kumi, Patrick Mensah-Amoah, Samuel Sonko Sackey

**Affiliations:** 1 Department of Physics, University of Cape Coast, PMB, Ghana; 2 Department of Agricultural Engineering, University of Cape Coast, PMB, Ghana

**Keywords:** porous substrate, polyurethane foam, image analysis, pore morphology, inexpensive imaging systems

## Abstract

This study investigated the pore characterization of polyurethane (PU) foam as a necessary step in water filtration membrane fabrication. Porous material characterization is essential for predicting membrane performance, strength, durability, surface feel, and to understand the transport mechanisms using modeling and simulations. Most existing pore characterization techniques are relatively costly, time-consuming, subjective, and have cumbersome sample preparations. This study focused on using three relatively inexpensive imaging systems: a black box, Canon camera (EOS760D), and LaserJet scanner (M1132 MFP). Two standard, state-of-the-art imaging systems were used for comparison: a stereomicroscope and a scanning electron microscope. Digital images produced by the imaging systems were used with a MATLAB algorithm to determine the surface porosity, pore area, and shape factor of the polyurethane foam in an efficient manner. The results obtained established the compatibility of the image analysis algorithm with the imaging systems. The black box results were found to be more comparable to both the stereomicroscope and SEM systems than those of the Canon camera and scanner imaging systems. Indeed, the current research effort demonstrates the possibility of substrate characterization with inexpensive imaging systems.

## 1. Introduction

Globally, the production of polyurethane (PU) foams dominates all other polymeric foams [1] as they are commercially inexpensive. Mostly, polymeric foams are the first choice for a wide range of technological applications, and PU foam consumption is 50% of the global polyurethane consumption [2]. The processing technology required to produce them is well-established when compared with foams derived from other polymers [2]. PU foams are lightweight, excellent porous materials with tunable thermal stability plus mechanical and sound-absorption properties for a range of applications, suitable for new and advanced technologies [1]. PU foams are generally made up of polyols and isocyanates. They can be recycled or recovered after use to reduce production costs and increase material utilization efficiency [3]. Thermosetting polymers like PU foams are widely preferred due to their light weight, excellent biocompatibility, flexible mechanical properties, and the presence of a urethane linkage (–NH–CO–O–) that bonds easily with nanoparticles such as silver nanoparticles (AgNPs) [4,5,6,7,8,9,10]. Also, PU foams have the capability of delivering a wide range of cell structures, rigidity, strength, and durability. The cell structure, whether it is open or closed, is another variable that must be considered. However, most flexible foams are designed to be of an open cell structure type, excellent for water and oil filtration research [11,12]. Rigid PU foams are mostly preferred in sandwich panels due to their high stiffness. They serve as shock absorbers when used in packaging and cushioning [12,13]. The cushioning properties improve when particles are added, and PU foams comply very much with fabrics and the incorporation of textile wastes [14]. Other distinguishing features of PU foams include high porosity and crushability [15]. Therefore, the properties plus technical and economic factors decide the effective use of PU foams.

Generally, porous materials have numerous applications in the fields of petroleum engineering, powder technology, membrane distillation processes, mass and heat transfer as well as in the fuel cell industry, and for the fabrication of cushion composites [12,16,17,18,19,20]. Membrane-based filtration remains a common technique in the water and oil treatment industries [21,22,23] because of the use of inexpensive PU foams, since the use of porous materials as substrates for membrane fabrication is always the preferred technique [21]. In membrane filtration, the pore morphology of substrates determines the permeate particle size, fouling rate, flux, and flow rate of a fabricated membrane [24,25]. Hence, understanding the pore morphological properties, such as pore area, shape factor, and porosity of a substrate, is a necessary step in approximating the efficiency and lifetime (fouling resistance) of filtration membranes [6].

Many publications [4,6,12,21,23,24,25] have demonstrated that PU foams exhibit high stability against chemical degradation, high mechanical durability, significant cushion properties, good swelling behavior, ease of separation from a solution, and are also some of the most inexpensive available polymer products. Several two-dimensional, relatively expensive, low-throughput, and complex imaging tools have been used for porous material characterization including optical microscopy, scanning electron microscopy (SEM), field emission SEM (FESEM), and transmission electron microscopy (TEM) [26]. She et al. [25] reported the pore morphological characterization of porous polyvinylidene fluoride or polyvinylidene difluoride (PVDF) membranes using SEM images, with much emphasis on the effective pore diameter. Furthermore, Datta et al. [27] employed an accurate and simple method of determining the porosity of different rock samples using image analysis. Recent studies have also shown the use of TEM to analyze CuNP-modified PU foams [28]. Abramoff et al. [29] reported the determination of pore sizes, pore size distribution, and other pore morphologies using FIJI/ImageJ from scanning electron microscope micrographs and a JEOL Model JSM-6490. A study by Rastegarfar et al. showed the characterization of PU foams produced from liquefied sawdust using SEM, among other techniques [8]. Bachtiar et al. recently investigated the thermal stability and fire resistance of flax fabric-reinforced polymer composites with the help of SEM [30]. However, most of the imaging tools employed by these researchers are relatively costly, time-consuming, and subjective, with cumbersome sample preparation and inconsistent results for materials with larger pores [6,26].

In recent times, optical flatbed scanners and digital cameras that employ charge-coupled devices (CCD) as sensors are being used as imaging systems for the morphological characterization of porous materials. These high-throughput systems are inexpensive, need little to no sample preparation, and serve as better alternatives [25,26,31]. Their acquired images are almost always processed using image processing software such as ImageJ, MATLAB, ICY, Avizo, and Image-Pro [26]. Although image analysis techniques are being employed by most researchers for the morphological characterization of porous materials, our literature search indicated that little attention has been given to the use of inexpensive imaging systems and devices with image analysis as a means of characterizing PU foams. 

In applications like adsorption and the permeability of porous substrates, material′s morphological properties, such as porosity, determines its applicability in technologies like membrane filtration. Thus, the specific objective of this proof-of-principle study was to use three inexpensive digital imaging systems/devices and two standard imaging systems to characterize the pore morphology of a PU foam. This was assisted by an image processing procedure in the MATLAB program to assess their comparability and show the significance of the proposed method for wider possible technological applications. 

## 2. Theory

The porosity (∅) of a porous material is defined as the ratio of pore volume ($V_{P}$) to the total volume ($V_{T}$) of the material. It can be expressed as a fraction (Equation (1)) or percentage (Equation (2)) [25,26]:

(1) $$\emptyset =\frac{V_{P}}{V_{T}}$$

(2) $$\emptyset =\frac{V_{P}}{V_{T}}\times 100$$

For the current study, the surface porosity became the ratio of the number of white pixels (Px) to the total area of the membrane surface (*L* × *W*), as in Equation (3) [25],

(3) $$\mathrm{Porosity}\;\left(\rho \right)=\frac{n_{w}}{L\times W}$$

where *L* and *W* represent the length and width of the cropped PU foam image (Figure 2), respectively [25]. The shape factor ($S_{F}$) is another very important morphological parameter which depends on the pore area (*A*) and the pore perimeter (*P*). It indicates the behavior of the material [32]. It also numerically determines the degree of circularity of the pores and is defined as in Equation (4) [17]:

(4) $$S_{F}=\frac{P^{2}}{4\pi A}$$

## 3. Materials and Methods 

**Figure 1:**
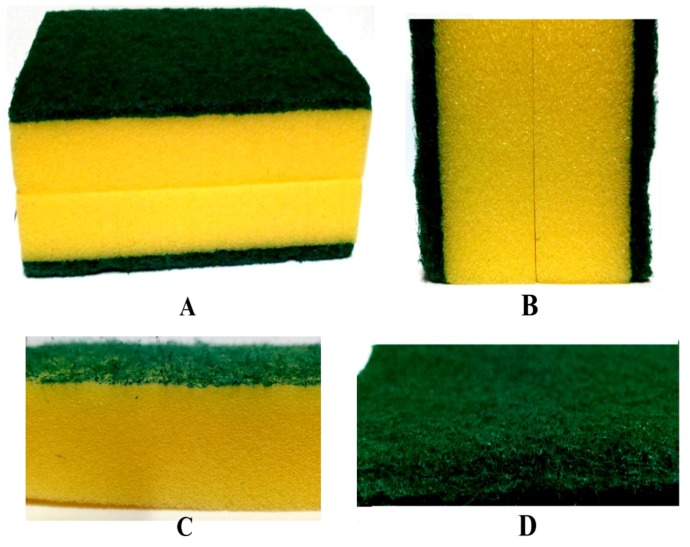
Commercially available polyurethane (PU) foam: (**A**) full sample purchased, (**B**) cross section of A, (**C**) cross section of core PU foam (yellow), and (**D**) investigated sample (green PU foam).

**Figure 2:**
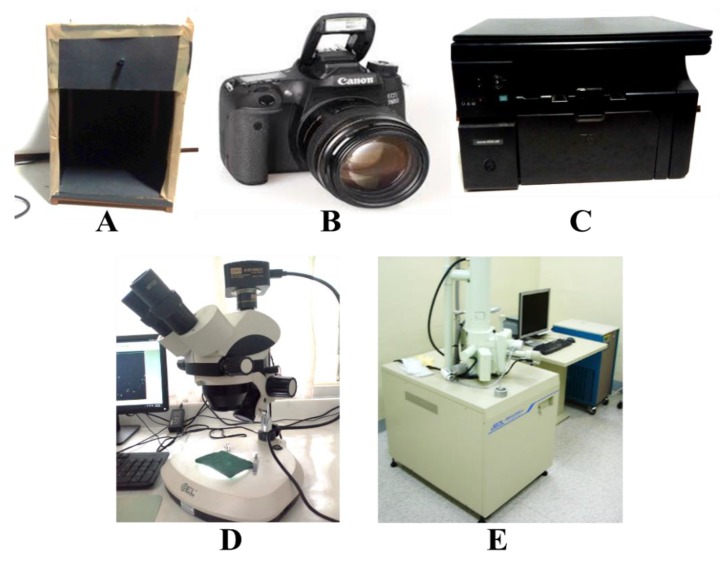
Android mobile phone photographs of the digital imaging systems/devices: (**A**) reflectance-based black box, (**B**) Canon camera (EOS 760D), (**C**) LaserJet scanner (M1132 MFP), (**D**) scanning electron microscope (JOEL Co. Ltd., Peabody, MA, USA, and (**E**) stereo microscope (BEL Photonics, Sao Paulo, Brazil).

Commercially available PU foam samples (15 cm × 7 cm; Figure 1A) were purchased from a local market in Ghana, and the green section (Figure 1B) ripped off from the sandwiched core PU foam was used for the study. The green section was more rigid than the sandwiched core. Figure 1B was investigated using three inexpensive digital systems/devices (Figure 2): a reflectance-based black box with a white light source (Figure 2A); an EOS 760D Canon camera (resolution of 6024 × 4022) (Tianjin, China); an RGB color filter array; an EFS range of 18–50 mm (Figure 2B); and an HP LaserJet scanner (M1132 MFP, Figure 2C) (HP development company, Palo Alto, CA, USA). In addition, two standard imaging systems (relatively expensive) were also employed to establish the comparability with the inexpensive imaging systems/devices: a stereomicroscope (BEL Photonics, Sao Paulo, Brazil) (Figure 2D), with a magnification of 1.8×; and a JSM-6390LV scanning electron microscope (JOEL Co. Ltd., Peabody, MA, USA), with a scanning voltage of 10 kV and magnification of 30× (Figure 2E). The reflectance-based black box with a white light source was made at the Laser and Fibre Optics Centre (LAFOC), Department of Physics, University of Cape Coast, Ghana. The black box was entirely black inside and outside, with a camera (webcam) and a white light source placed at the top of the rectangular box (39 mm × 23.5 mm).

In this study, the following steps show how the images were acquired, processed, and analyzed (with appropriate MATLAB commands added).

**Step 1:** Five images were acquired for each imaging system and saved in TIF format. Each image was selected, read, and cropped to a size of 450 × 450 using the command below: 

Command: [fname,path] = uigetfile(“.”,′SELECT AN IMAGE′);

fname = strcat(path,fname); 

I = imread(fname);

Icrop = imcrop(I);

**Step 2:** The cropped input images were converted to grayscale using the following command:

Command: Igray = rgb2gray (Icrop);

**Step 3:** The image intensity of each grayscale image was adjusted using histogram equalization and median filtering: 

Command: Ia_eq = adapthisteq (Igray);

Ifiltered = medfilt2 (Ia_eq, [3,3]);

**Step 4:** Image segmentation was done using adaptive thresholding with the following command: Command: Ibin = blkproc (Ifiltered, [15,15], @adaptt);

**Step 5:** Each segmented image was then complemented using the command below:

Command: Icomp = imcomplement (Ibin);

**Step 6:** The complemented binary image dimensions (length width) were determined as [X Y], and the number of black and white pixels calculated as “nblack (nb)” and “nwhite (nw)”. The surface porosity was then calculated for each image using Equation (3): 

Command: [X,Y] = size (Icomp);

nBlack = sum(Icomp (:));

nWhite = numel (Icomp) − nBlack;

Porosity = nWhite/(X*Y);

**Step 7:** Closing and opening morphological operations were performed on each complemented image using a disk structuring element. The surface porosity was calculated again after each of these operations:

Command: Se = strel (′disk′,3);

Iclose = imclose (Icomp,Se);

Iopen = imopen (Icomp,Se);

**Step 8:** The connected components in each image (complemented, closed, and opened) were identified. The pore regions (i.e., a pore structure here is used to describe the porosity, pore size, pore size distribution, and pore morphology of a porous medium) were assigned with unique labels and used in calculating the pore parameters in each image. The average values of each parameter were exported in Microsoft (MS) Excel and their corresponding standard deviations calculated. The shape factor (S_Factor) was calculated using Equation (4). All the dimensional parameters were measured in pixels (Px).

Command: Icc = bwconncomp(Icomp); 

S_Factor2 = (Perimeter2^2)/(4*pi*Area2); 

**Step 9:** The pore area distribution for each image was also plotted to indicate the frequency of larger pores versus smaller pores on the PU surface.

**Figure 3:**
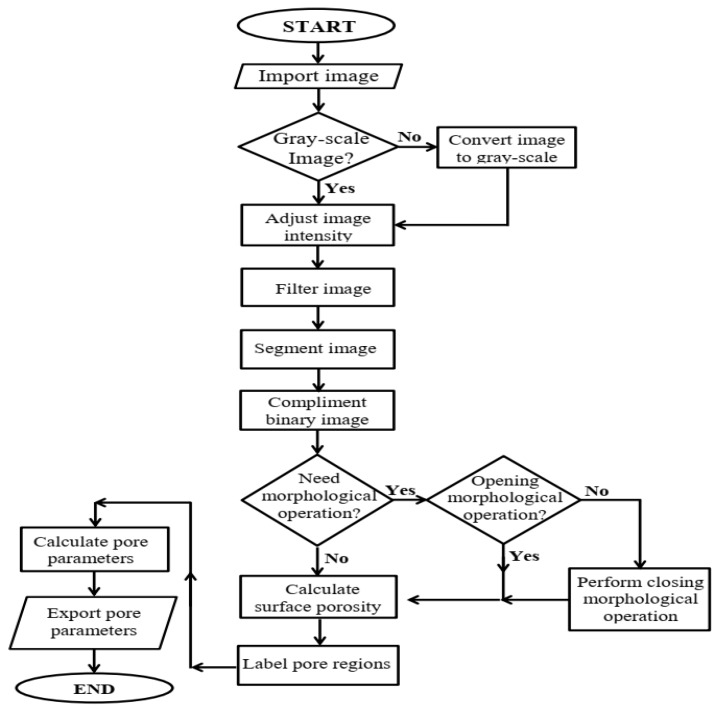
Algorithm of image processing and analyses executed in the MATLAB 2017a.

Syntax: A = [stats.Area];

bar(A,8);

The flowchart (Figure 3) outlines the algorithm of the image processing and analyses executed in the MATLAB 2017a program for images obtained with each imaging system. 

## 4. Results and Discussion

**Figure 4:**
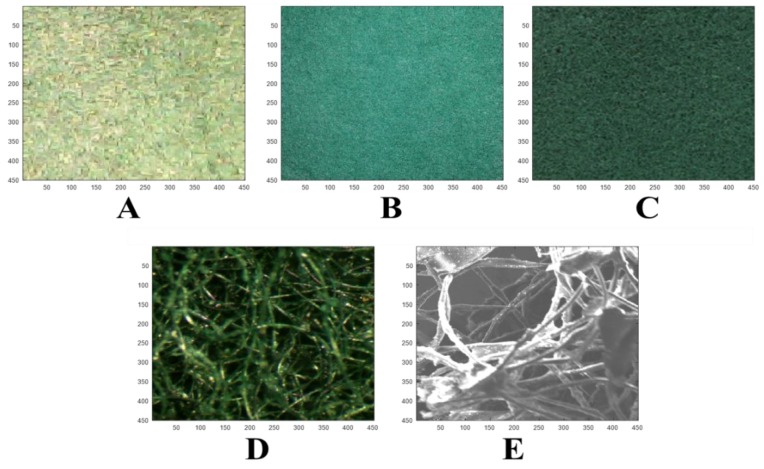
Scaled input images for (**A**) black box, (**B**) Canon camera, (**C**) scanner, (**D**) stereomicroscope (magnification = 1.8X), and (**E**) scanning electron microscope (magnification = 30×; scale = 500 µm).

The green section (Figure 1B) shows the investigated sample, with the cropped and scaled input images for the imaging systems in Figure 4A–E. The cropped images obtained with the imaging systems/devices showed very clear differences in terms of the surface morphology, color, and contrast. In particular, the stereomicroscope and SEM images (Figure 4D,E) showed well-defined fiber structures (e.g., open cell) due to their high resolutions and magnifications of the imaging systems. 

**Figure 5:**
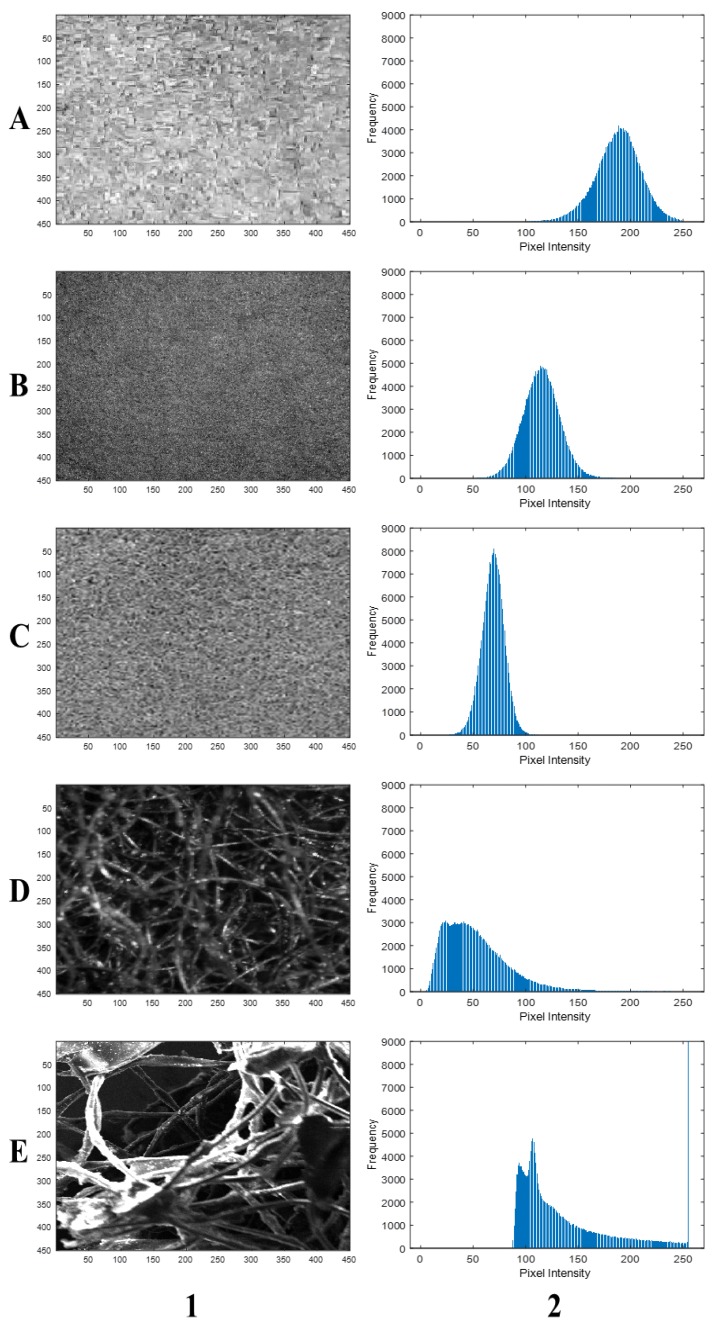
Scaled grayscale images (**1**) and histograms (**2**) by (**A**) black box, (**B**) Canon camera, (**C**) scanner, (**D**) stereomicroscope, and (**E**) scanning electron microscope.

**Figure 6:**
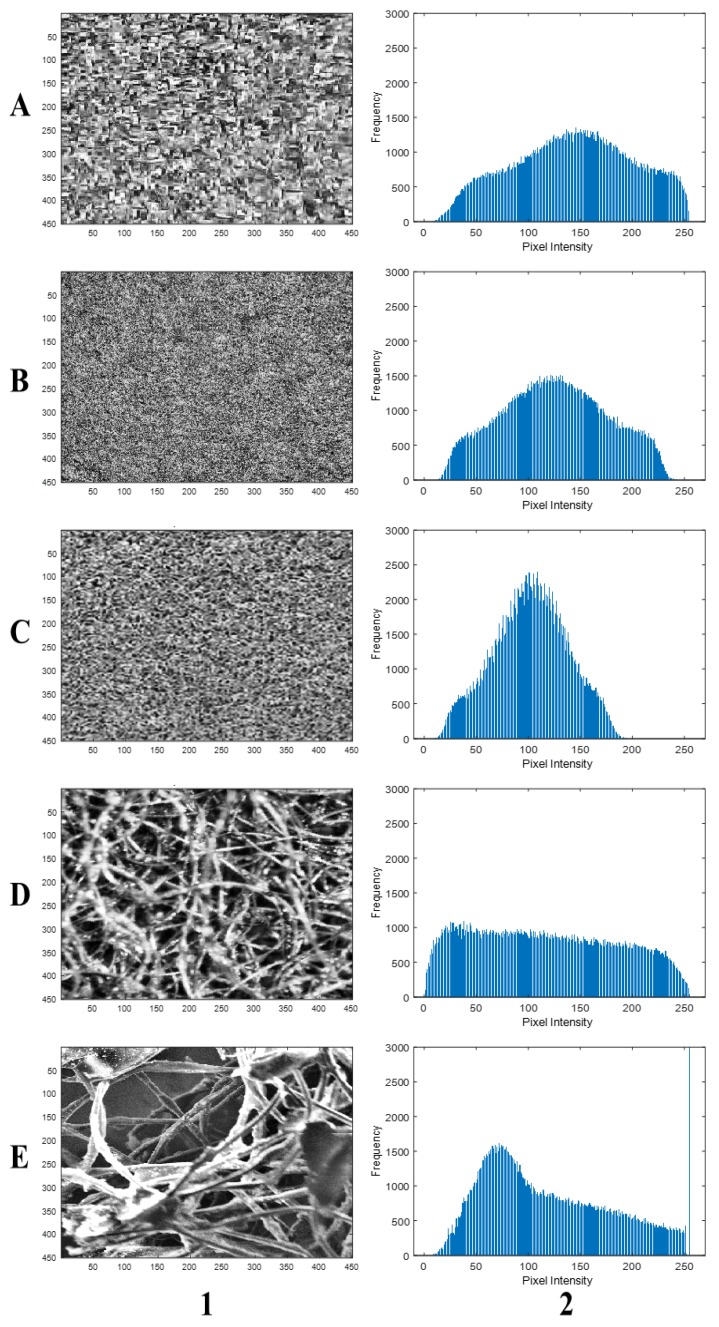
Scaled equalized images (**1**) and histograms (**2**) for (**A**) black box, (**B**) Canon camera, (**C**) scanner, (**D**) stereomicroscope, and (**E**) scanning electron microscope.

**Figure 7:**
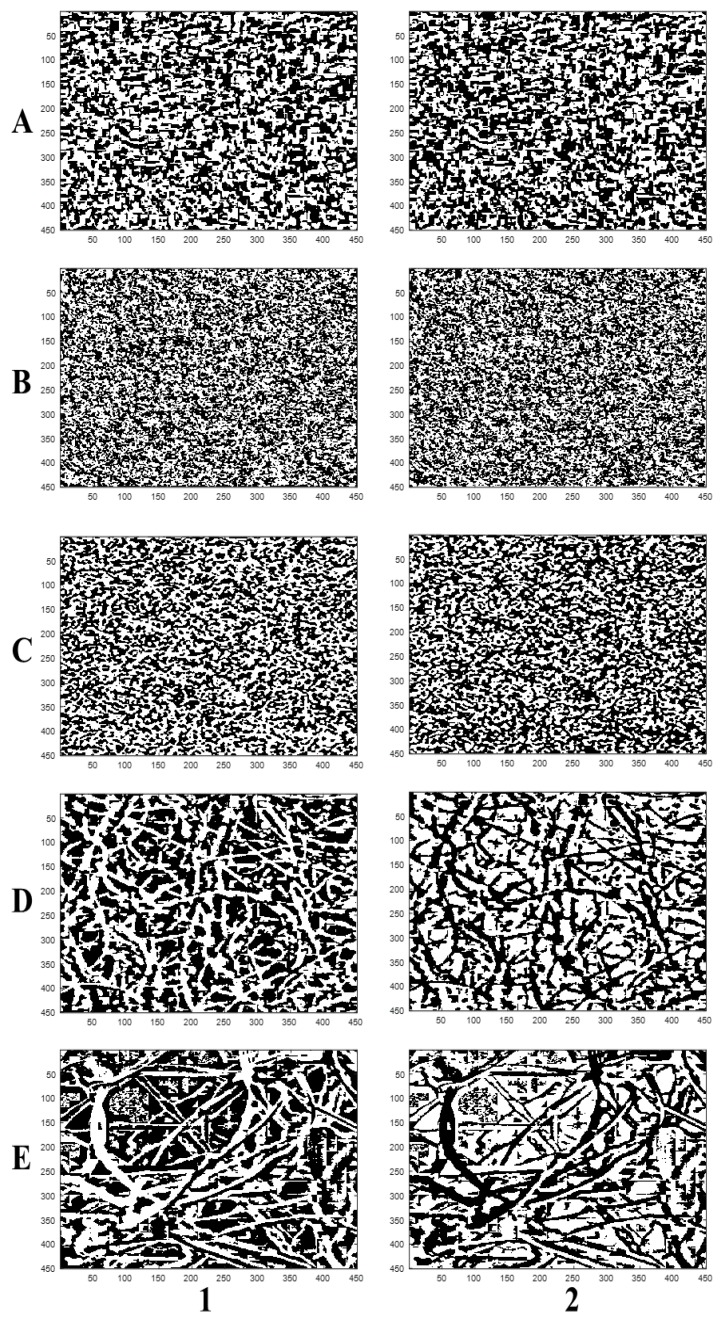
Scaled binary (**1**) and complemented (**2**) images for (**A**) black box, (**B**) Canon camera; (**C**) scanner, (**D**) stereomicroscope, and (**E**) scanning electron microscope.

Figure 5 represents the grayscale images with their corresponding histograms. These histograms showed the absence of highlight and shadow clipping, thus, underscoring no significant loss of image data. They were generally skewed in different directions. Nevertheless, those of the black box and SEM (Figure 5(A-2,E-2)) skewed more into the highlight region and showed significant similarities and differences between the imaging systems. Furthermore, the equalized images and their histograms (Figure 6), as well as binary and complemented images (Figure 7), showed similar characteristics among the imaging systems. In general, the micrographs showed open cell structures of the PU foam used and demonstrate the manifold uses of PU foams.

The histogram equalization was applied to improve the contrast [33], and also for uniform intensity distribution. The median filtering was also applied to remove noise in each image for better contrast. The intensity distribution of the equalized images obtained for the black box, stereomicroscope, and scanning electron microscope were more comparable. The stereomicroscope image, however, displayed a much more uniform distribution of pixels across the grayscale. The filtered images were segmented using adaptive thresholding to obtain the binary images and their complements (Figure 7). More conventional thresholding techniques, like Otsu′s method, use global thresholds for all pixels. With adaptive thresholding, the threshold value is changed over the entire image [34]. Therefore, the adaptive thresholding technique was employed to avoid significant loss of image data and improve accuracy. 

Moreover, Otsu′s thresholding technique could not deal with noisy images (like those from the black box) effectively and was only suitable for images with a bimodal intensity distribution [19,21,28,35]. The resulting binary images after segmentation were complemented to switch the foregrounds and backgrounds. The images by the black box exhibited morphological features (white area distribution) similar or closer to those by the stereomicroscope and SEM systems. To further demonstrate differences and similarities among the imaging systems, closing and opening morphological operations were applied to smooth the pore objects and remove isolated noise pixels, which helped to improve the contrast between the images. However, a significant loss of image data through erosion and dilation with the opening and closing morphological operations may have affected the accuracy of the calculated parameters [36]. To this end, Table 1 shows the mean values of the surface porosity, pore area, and shape factor obtained by each imaging system with their respective standard deviations.

**Figure 8:**
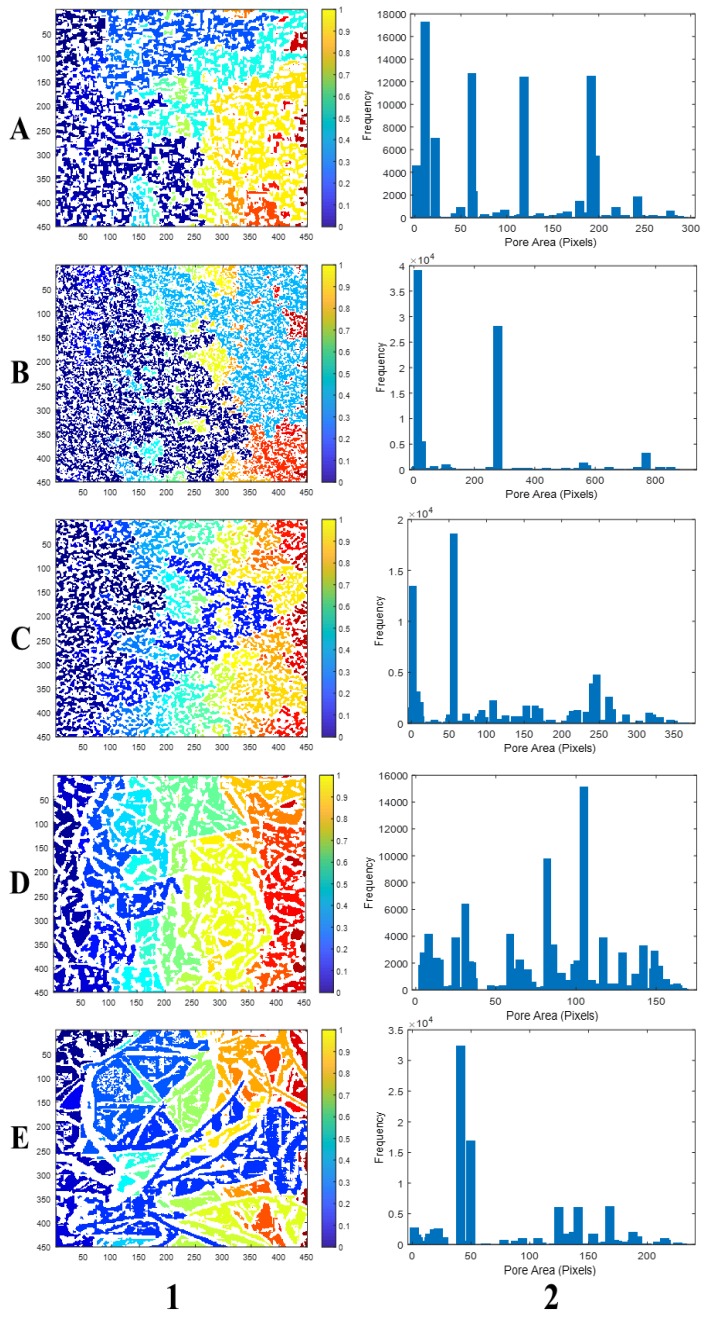
Scaled labeled images (**1**), and frequency distribution of pore area (**2**) for: (**A**) black box, (**B**) Canon camera, (**C**) scanner, (**D**) stereomicroscope, and (**E**) scanning electron microscope.

The surface porosities calculated on the complemented image (Icomp), closed image (Iclose), and opened image (Iopen) showed significant variations among the imaging systems. Nevertheless, the surface porosities on Icomp gave the lowest standard deviations for the black box and scanner. Hence, the computed morphological parameters of surface porosity, pore area, and shape factor were calculated based on Icomp for the five imaging systems for comparison. A bar chart plot of frequency distribution of the pore area (Figure 8) showed the number of large pores relative to small pores in the images by each of the imaging systems. All the imaging systems showed an inhomogeneous distribution of large and small pores across the PU foam, and this may be due to the type and functionality of the polyol or isocyanate used in the foaming process/manufacturing [1].

The assumption could be that the distribution in pore size were either Maxwellian or Gaussian. However, in some porous materials, other types of distribution may occur. In fact, this may be dependent on the pore model, shape factor, and the thickness of the porous material. For example, the pore distribution according to the stereomicroscope image shows a slightly even distribution of the different pore sizes (Figure 8(D-2)), and was Gaussian. On the other hand, the pore area distributions by the SEM were Maxwellian to a large extent. This might be due to the uniform grouping of the same color-labeled pores at particular sections in the labeled image (Figure 8(D-1)), which was not the case in the other images (Figure 8(A-1,B-1,C-1,E-1)). Comparatively, the pore distribution by the black box was much closer to that of the SEM, mostly in the small size regime. In general, the imaging ability of pores by the inexpensive imaging devices was best with the black box, and very comparable with the pore distributions obtained with the SEM and stereomicroscope (Figure 8). 

The calculated pore parameters for the black box images were found to be more comparable with those of the microscope and SEM under Icomp (Table 1). Olson [37] showed that shape factor is an important parameter in characterizing porous materials with very irregular pores. It is dependent on the pore area and perimeter. In this study, the presence of irregularly shaped pores in the PU foam is evident. Moreover, the variation in the pore area as shown in Table 1 may be due to the differences in the imaging devices, their operating resolutions, and magnifications. Therefore, pore characteristic features can be estimated from the microstructure of PU foams [38]. Accordingly, Lurosso et al. [39] successfully characterized the thermophysical and mechanical properties of PU foams using SEM images. Thus, scanning electron microscopes are regarded as a standard imaging tool for morphological studies of PU foams. Nevertheless, Ariff et al. [40] noted that although scanning electron microscopes are effective, they tend to be expensive. 

## 5. Conclusions

In this study, an image analysis algorithm was successfully used in the morphological characterization of PU foams using inexpensive digital imaging systems in comparison with the more expensive and standard scanning electron microscopes and stereomicroscopes. The inexpensive devices are simple, easy to use with high-throughput performance, reliable, and accurate. In particular, the use of the imaging black box system showed the potentiality in using relatively low-cost smartphones for significant accuracy in the pore morphological characterization of porous materials in numerous applications including nanotechnology applicability in water–oil remediation, petroleum engineering, powder technology, sound-proofing technology, and cushioning technology. In fact, PU foams have become popular in everyday life products and represent the most important class of polymers that can change the quality of human life [1]. The computed morphological parameters of surface porosity, pore area, and shape factor are very essential in the substrate selection for advanced technologies like the fabrication of filtration membranes. They also influence the effectiveness of the porous substrates in performance prediction, strength, and durability in water and/or oil filtration systems. Generally, the study revealed that the pore diameter of PU foams is usually big enough so that the pores of PU foams can be observed by the naked eye. Therefore, it can be quantitatively analyzed by imaging devices without high magnification, e.g., the black box and some smartphones. Thus, using such cost-effective imaging tools to study the pore structure of polyurethane foams is of great significance. To a large extent, the present study showed that using an economically inexpensive digital imaging device/system and image analysis is novel and a surrogate method for accurate determination of the morphological characterization of porous materials (e.g., PU foams). Apart from a few outliers, the presented algorithm is simple and fast (run time of 16.08 s) with an appreciable level of accuracy.

## Figures and Tables

**Table 1:** Computed pore parameters for imaging systems.

	Black Box			Canon Camera			LaserJet Scanner			Microscope			SEM		
	Surface Porosity (1)	Pore Area (Px)	Shape Factor (1)	Surface Porosity (1)	Pore Area (Px)	Shape Factor (1)	Surface Porosity (1)	Pore Area (Px)	Shape Factor (1)	Surface Porosity (1)	Pore Area (Px)	Shape Factor (1)	Surface Porosity (1)	Pore Area (Px)	Shape Factor (1)
**I_comp_**	0.515 ± 0.013	342.59 ± 69.62	1.64 ± 0.42	0.579 ± 0.138	124.17 ± 18.02	1.91 ± 0.06	0.546 ± 0.007	75.49 ± 10.71	1.80 ± 0.07	0.455 ± 0.014	845.71 ± 175.06	2.99 ± 0.29	0.450 ± 0.017	449.71 ± 127.08	1.73 ± 0.35
**I_closed_**	0.366 ± 0.031	5203.94 ± 1519.66	0.34 ± 0.06	0.206 ± 0.037	9372.22 ± 5691.92	0.31 ± 0.14	0.212 ± 0.026	3520.18 ± 986.77	0.13 ± 0.07	0.387 ± 0.013	8197.14 ± 2546.39	2.69 ± 0.48	0.340 ± 0.028	6704.29 ± 3205.38	1.16 ± 0.66
**I_open_**	0.666 ± 0.036	166.21 ± 43.97	1.38 ± 0.16	0.829 ± 0.025	64.20 ± 9.18	0.99 ± 0.04	0.862 ± 0.039	56.45 ± 10.04	0.93 ± 0.05	0.512 ± 0.017	498.28 ± 107.14	1.92 ± 0.22	0.534 ± 0.022	421.71 ± 57.10	1.76 ± 0.17
